# Comparative Pathways of SO_2_ Formation on the Pyrite (100) Surface: A DFT Study of O_2_ Activation and Surface Sulfur Oxidation

**DOI:** 10.1021/acsomega.6c06241

**Published:** 2026-10-23

**Authors:** Mingrui Liu, Hongtao Li, Xiqing Xu, Jie Sha, Guangyuan Xie

**Affiliations:** † Key Laboratory of Coal Processing and Efficient Utilization of Ministry of Education, School of Chemical Engineering and Technology, 12392 China University of Mining and Technology, Xuzhou 221116, China; ‡ Chifeng Disaster Prevention and Relief Center, Chifeng 024000, China; § State Key Laboratory of Continental Shale Oil, Daqing 163712, China

## Abstract

The microscopic mechanism of SO_2_ formation from pyrite (the dominant inorganic sulfur species in coal) remains unclear, particularly the comparative energetics and relative competitiveness of pathways from O_2_ activation to SO_2_ formation and desorption. This knowledge gap limits the development of predictive models for pyritic sulfur conversion during coal combustion. In this work, density functional theory (DFT) calculations were used to examine a reaction network for pyrite oxidation on the FeS_2_ (100) surface. Multiple adsorption, dissociation, readsorption, precursor-formation, and desorption pathways were compared using LST/QST-derived transition-state candidates and consistent single-point energy calculations. Stage-wise comparison of the examined channels supports 3→A→V→V-(2) as a representative candidate sequence. For this sequence, the estimated local barrier for SO_2_ precursor formation is 119.28 kJ/mol, which is the lowest among the six precursor-formation routes examined. Compared with II-(2), the V-(2) transition-state candidate exhibits a smaller S displacement while retaining local Fe–O coordination.

## Graphical abstract

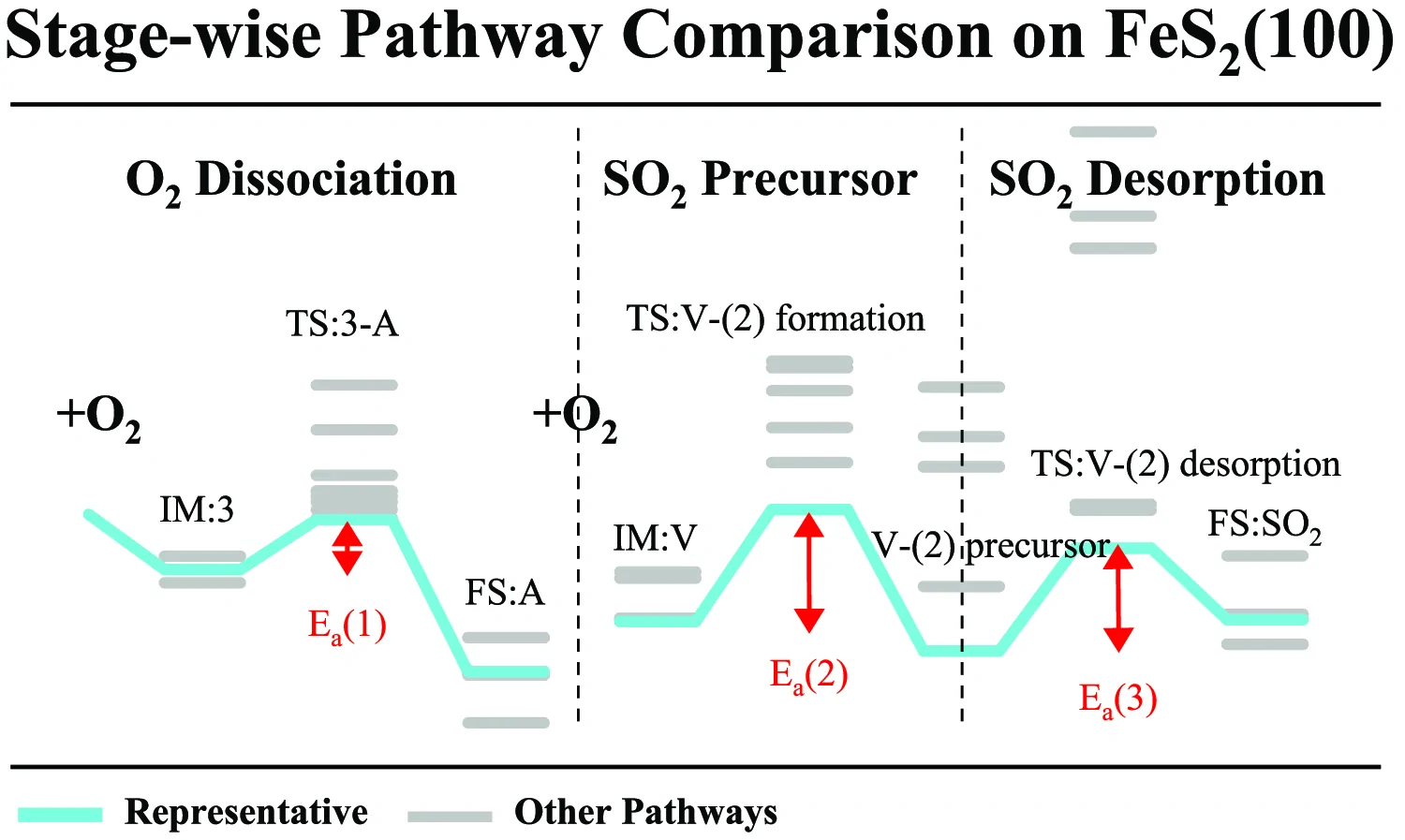

## 1 Introduction

Pyrite (FeS_2_) is the dominant inorganic sulfur species in coal.^1–3^ Its oxidation is relevant to acid mine drainage and the weathering of pyrite-bearing materials, as well as to SO_2_ formation and ash-related behavior during coal combustion.^4–19^ Atomic-scale analysis is therefore needed to resolve the elementary steps of the activation of O_2_ and sulfur oxidation.

Experimental studies have provided a comprehensive understanding at the macroscopic level of pyrite’s transformation pathways and product distributions in air or oxygen-rich environments, as well as the staged characteristics of its oxidation and the release profiles of gaseous products.^1,20–30^ However, due to the limited capability of experimental techniques to probe processes at the atomic scale, key scientific questions, including the identification of elementary steps, quantification of reaction barriers, and clarification of S/O migration pathways, still require further theoretical investigation.

Regarding surface structural stability and reconstruction, existing studies show that pyrite exhibits significant differences in stability across crystallographic facets, and that surface relaxation and defects play a critical role in determining this stability. Mehlape et al.^31^ reported the surface energy ordering as (100) < (111) < (110) < (210). Xian et al.^32^ obtained the surface-energy order (100) < (210) < (110) < (111) for stoichiometric pyrite surfaces using PBE+*U*; under sulfur-poor and sulfur-rich conditions, the system preferentially forms (100)-S and (111)-3S terminated surfaces, respectively. Their work also demonstrated that during surface relaxation and reconstruction, electrons transfer from surface Fe atoms to S atoms. Moreover, Xian et al.^33^ revealed a general relaxation trend in pyrite surfaces: top-layer S atoms move outward while Fe atoms relax inward, leading to a sulfur-rich (S-rich) surface. Chen et al.^34^ further showed that under Fe-deficient conditions, S*_n_* polymeric species form on the surface; these exhibit weak adhesion to the substrate but high cohesive energy, thereby inhibiting oxidation. In contrast, under S-deficient conditions, [Fe–S]*_n_* chain-like structures develop, which bind strongly to the substrate but possess low cohesive energy, consequently accelerating surface oxidation.

Regarding the mechanism of O_2_ adsorption and activation, current theoretical studies reveal multiple coexisting pathways. Sacchi et al.^35^ found that O_2_, N_2_, NO, and NO_2_ molecules chemisorb predominantly on surface Fe sites, while S sites show negligible reactivity and even suppress NO_2_ adsorption at Fe–Fe bridge sites. Sun et al.^36^ similarly observed electron transfer from Fe to S atoms and reported that side-on O_2_ adsorption is more favorable than end-on adsorption, with an elongated O–O bond length characteristic of a peroxo-like state. Li et al.^37^ demonstrate that O_2_ dissociative adsorption on the pyrite (100) surface can occur at both Fe–S and S–S sites, with the strongest adsorption energy (approximately – 280 kJ/mol) at the S–S site. Rozgonyi et al.^38^ further showed that on a clean surface, O_2_ preferentially adsorbs in a side-on configuration across adjacent Fe sites and, upon dissociation, primarily oxidizes surface S atoms. In contrast, at sulfur vacancies, O_2_ forms a stable peroxo complex, and adsorbed S atoms are readily oxidized to SO_2_ and desorbed – highlighting the high reactivity of surface defects. Sit et al.,^39^ using DFT, confirmed that O_2_ initially adsorbs on the pyrite (100) surface, favoring a side-on configuration bridging two adjacent Fe atoms. Cleavage of the O–O bond in this configuration has a low activation barrier (only 25.5 kJ/mol) and is highly exothermic (−109.6 kJ/mol), facilitating subsequent oxidation steps.

Yang et al.^40^ proposed the detailed mechanism of SO_*x*_ generation. They reported that the surface oxidation stage mainly proceeds through three steps: oxidation of surface lattice sulfur, SO_2_ desorption, and sulfur vacancy healing with the oxidation of lattice sulfur as the rate-limiting step. In the bulk oxidation stage, four steps are involved: sulfur migration, oxidation, desorption, and oxygen deposition, where sulfur migration is the rate-limiting step and the SO_2_ formation pathway is more favorable than that of SO_3_.

Building on the multistep SO*_x_*-formation framework proposed by Yang et al.,^40^ the present work compares alternative surface reaction channels on FeS_2_(100), including three O_2_ adsorption configurations, nine O–O dissociation pathways, six O_2_ readsorption configurations, six SO_2_-precursor-formation pathways, and the corresponding SO_2_ desorption processes.

The representative sequence was assembled by considering estimated activation barriers, intermediate energies, and the structural correspondence between successive stages. Accordingly, 3→A→V→V-(2) is discussed as a representative candidate sequence for the possible evolution from O_2_ activation to SO_2_ precursor formation and release. This study therefore extends the previous mechanistic framework by comparing the energetics and structures of competing channels.

## 2 Computational Methods

First-principles calculations^41,42^ were performed based on density functional theory (DFT). The GGA-PBE^43^ functional was combined with the Tkatchenko-Scheffler dispersion correction^44^ (DFT-D) to account for exchange-correlation and van der Waals interactions. Ultrasoft pseudopotentials (OTFG ultrasoft) were used to describe the interactions between ionic cores and valence electrons. Spin-polarized DFT was used for adsorption geometry optimizations, isolated O_2_ reference calculations, and single-point energy and population analyses. The LST/QST transition-state searches were performed without spin polarization. This approach has proven effective in describing adsorption behavior and charge transfer characteristics at sulfide mineral surfaces.^33–35,37^ A plane-wave cutoff energy of 571.40 eV was used for bulk calculations, geometry optimizations, and single-point energy evaluation, whereas 450.00 eV was used only for the LST/QST pathway searches. A cutoff-energy sensitivity test over 450–700 eV showed progressively smaller changes above 550 eV; the CASTEP Fine setting of 571.40 eV was therefore adopted as a balance between numerical accuracy and computational cost. The detailed cutoff-energy results are provided in the Supporting Information. The calculated lattice parameter was 5.374 Å, differing by less than 1% from the experimental value of 5.416 Å.

The (100) surface, which has the lowest surface energy and is easily exposed to the environment,^40,45^ was selected as the model substrate. Compared with Yang et al.,^40^ the present calculations use the same FeS_2_(100) surface orientation and PBE-based CASTEP framework, but differ in several computational details. Yang et al.^40^ used a periodic (2 × 2) FeS_2_(100) slab with 9 atomic layers, a 20 Å vacuum region, a 280 eV plane-wave cutoff, a 2 × 2 × 1 k-point mesh, and fixed the bottom six layers while relaxing the top three layers. In contrast, the present work uses a periodic (2 × 1 × 1) supercell slab with 9 atomic layers (36 atoms in total), a 15 Å vacuum layer, a 571.40 eV cutoff for optimized structures and single-point energies, a 450.00 eV cutoff for LST/QST searches, a 2 × 4 × 1 k-point mesh and Tkatchenko–Scheffler dispersion correction. Geometry optimizations and single-point energy evaluations were spin-polarized. These differences should be considered when comparing absolute barrier values between the two studies.

The bottom three atomic layers were held fixed, while the upper six layers were relaxed. The Brillouin zone was sampled using a 2 × 4 × 1 Monkhorst–Pack k-point grid, selected to maintain an approximately consistent reciprocal-space sampling density for the 2 × 1 surface supercell. Geometry optimizations were carried out using the BFGS algorithm with convergence criteria of 1.0 × 10^–5^ eV/atom for energy, 0.02 eV/Å for maximum forces, and 0.001 Å for maximum displacements. The self-consistent field (SCF) calculations were converged to 1.0 × 10^–6^ eV/atom. Transition-state candidates were identified using the LST/QST method. Because full vibrational-frequency and intrinsic-reaction-coordinate analyses were not performed, the located structures are described as transition-state candidates and the corresponding barriers are described as estimated comparative barriers.

The DOS and PDOS were evaluated for the three optimized O_2_ adsorption configurations using a smearing width of 0.20 eV and an energy-grid density of 100 points eV^–1^. The PDOS was projected onto the 3d states of the O_2_-coordinated surface Fe atom and the combined 2p states of the two adsorbed O atoms with the Fermi level set to 0 eV.

Fixed-geometry single-point population analyses were performed for the readsorbed Configuration V (initial state) and the SO_2_-separated final state of the V-(2) pathway. Both models contained the same neutral Fe_12_S_24_O_4_ periodic supercell and were evaluated using identical computational settings: PBE with Tkatchenko–Scheffler dispersion correction, OTFG ultrasoft pseudopotential, a plane-wave cutoff energy of 571.40 eV, a 2 × 4 × 1 k-point grid, spin-polarized calculation, and a total cell charge of zero. Mulliken charges, Hirshfeld charges, and bond populations were analyzed. The atomic coordinates and lattice parameters were kept fixed during these calculations. In the final-state model, the separated SO_2_ molecule remained within the vacuum region of the same periodic supercell. The population calculations at these two specific states were used to analyze electronic redistribution.

Adsorption energies were calculated using eq 1.^40,46^

1 $$E_{\left(\mathrm{ads}\right)}={E_{\left(\mathrm{pyrite}+O_{2}\right)}}^{\mathrm{Opt}}-\left({E_{\left(\mathrm{pyrite}\right)}}^{\mathrm{Opt}}+{E_{\left(O_{2}\right)}}^{\mathrm{Opt}}\right)$$

Here, *E*_(pyrite+O_2_)_^Opt^ denotes the ground-state total energy of the O_2_ molecule adsorbed on the pyrite (100) surface system after geometric optimization; *E*_(pyrite)_^Opt^ is the energy of the clean and relaxed pyrite (100) supercell surface; and *E*_(O_2_)_^Opt^ refers to the energy of the isolated O_2_ molecule optimized in the same vacuum supercell. For adsorption of the second O_2_ molecule, the preoxidized Configuration A was used as the surface reference in the adsorption-energy expression. All isolated O_2_ reference calculations used in the adsorption-energy expressions were spin-polarized and initialized with N↑–N↓ = 2, corresponding to *S* = 1 and a spin multiplicity of 3. The converged integrated spin density was 2.0 in each case, confirming that gas-phase O_2_ remained in its triplet ground state.

The estimated barrier *E*_a_ for each elementary step was calculated according to eq 2.^47^

2 $$E_{\left(a\right)}=E_{\left(\mathrm{transition}\right)}-E_{\left(\mathrm{intermediate}\right)}$$

where *E*_(transition)_ and *E*_(intermediate)_ represent the energy of the LST/QST-derived transition-state candidate and its preceding intermediate state, respectively. The reaction energy was calculated using eq 3.

3 $$\Delta E=E_{\left(\mathrm{final state}\right)}-E_{\left(\mathrm{intermediate}\right)}$$

where *E*_(final state)_ represents the energy of the final state. For each elementary step, *E*_a_ and Δ*E* are referenced to its initial state. Pathway comparisons were performed separately for each reaction stage. An overall energy profile referenced to a common initial state was not constructed, and no overall rate-limiting barrier was assigned.

## 3 Results and Discussion

### 3.1 Adsorption Characteristics and Activation Mechanism of O_2_ on the Pyrite (100) Surface

The adsorption and activation of O_2_ on pyrite surfaces constitute the initial step in pyrite oxidation during coal combustion, with the adsorption configuration, charge transfer characteristics, and bonding mode strongly influencing the energetic preference of subsequent reaction pathways. In this work, the pyrite (100) surface was selected as the substrate, and stable O_2_ adsorption configurations were systematically explored via geometry optimization across various active sites – including Fe, three-coordinated sulfur (S_3_c), four-coordinated sulfur (S_4_c), and hollow sites – and different adsorption orientations (end-on and side-on). The results show that, among all considered sites, only three stable adsorption configurations (Configurations 1–3) were identified, all of which involve bonding to Fe atoms. Under the structural search and computational settings employed in this work, no stable molecular O_2_ adsorption states were observed on the S-top, S-bridge, hollow sites, or other non-Fe sites. This finding is consistent with previous studies by Sacchi et al.,^35^ Rozgonyi et al.,^38^ and Sit et al.,^39^ confirming that Fe sites serve as the primary O_2_ adsorption and activation sites on the modeled pyrite (100) surface.

Notably, while Sun et al.^36^ and Sit et al.^39^ reported side-on O_2_ adsorption bridging adjacent Fe–Fe sites, the present study reveals that O_2_ can stably adsorb on a single Fe site in either side-on or end-on configurations. Key structural parameters of the three stable configurations are summarized in Figure 1.

**Figure 1:**
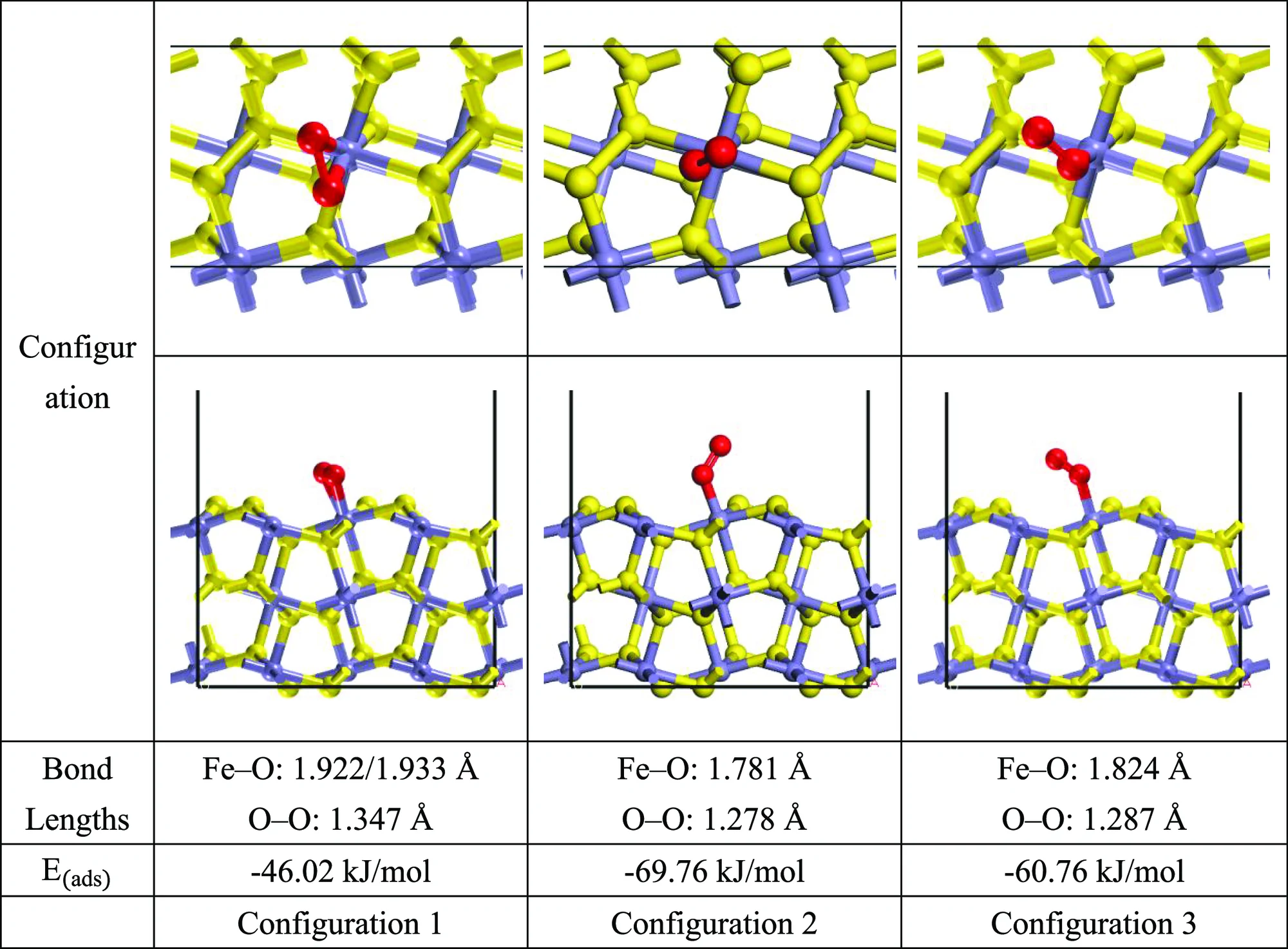
Three stable adsorption configurations of O_2_ molecules on the pyrite (100) surface with their bond lengths (unit: Å) and adsorption energies *E*_(ads)_ (unit: kJ/mol).

As shown in Figure 1, Configuration 2 is the most stable of the three initial adsorption configurations, exhibiting the most negative adsorption energy (−69.76 kJ/mol) and the shortest Fe–O bond length (1.781 Å).

In Configuration 1, the O_2_ molecule adsorbs side-on at a single surface Fe site, with both O atoms coordinated to the same Fe atom at distances of 1.922 and 1.933 Å. However, its adsorption energy (−46.02 kJ/mol) is less negative than that of Configuration 2, and the O–O bond is stretched to 1.347 Å (compared to 1.221 Å for gaseous O_2_), indicating stronger O–O activation despite its weaker overall adsorption.

The adsorption energy of Configuration 3 (−60.76 kJ/mol) is approximately 9.01 kJ/mol less negative than that of Configuration 2, and its Fe–O bond length (1.824 Å) differs only slightly from that of Configuration 2, suggesting that Configuration 3 may serve as another plausible precursor state.

Electron Density Difference (EDD) and Mulliken charge analysis were employed to investigate the electron transfer mechanisms and bonding characteristics of the three adsorption configurations. The EDD is defined as Δρ = ρ_(adsorption)_ – ρ_(surface)_ – ρ_(O_2_)_, where blue regions represent electron accumulation Δρ > 0 and red regions represent electron depletion Δρ < 0. The results are presented in Figure 2.

**Figure 2:**
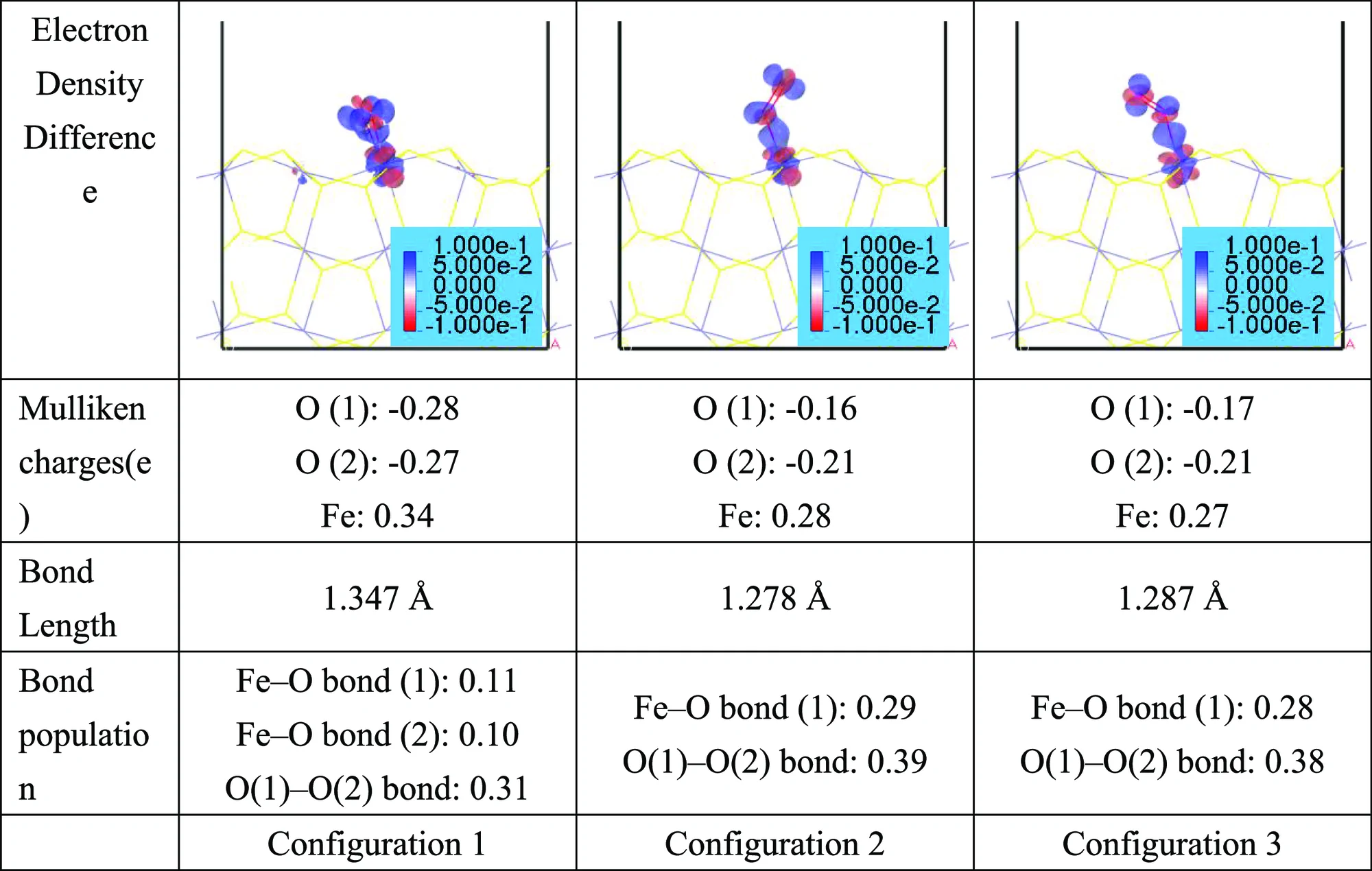
EDD and Mulliken charge analysis of the three O_2_ adsorption configurations on the pyrite (100) surface.

In Configuration 1, Mulliken charge analysis shows that the two oxygen atoms carry charges of −0.28e and −0.27e, respectively, while the coordinated surface Fe atom carries a charge of +0.34e. The two adsorbed O atoms carry a total Mulliken charge of −0.55e, consistent with electron uptake by O_2_ from the surface. The EDD map (Figure 2) shows the accompanying redistribution of electron density around the adsorbate and the coordinated Fe site. The similar charges on the two oxygen atoms suggest a nearly symmetric redistribution of electron density within the adsorbed molecule. The O–O bond is elongated from 1.221 Å in gas-phase O_2_ to 1.347 Å in Configuration 1, where the calculated O–O bond population is 0.31, further indicating activation and weakening of the O–O bond. In contrast, the Fe–O populations are only 0.11 and 0.10, respectively, indicating relatively weak Fe–O covalent interactions. This weak bonding nature is consistent with the less negative adsorption energy of −46.02 kJ/mol.

Configurations 2 and 3 adopt end-on adsorption geometries, leading to a more asymmetric charge distribution within the adsorbed O_2_ molecule. Mulliken charge analysis shows that the proximal oxygen atom, O(1), carries less negative charge (−0.16e and −0.17e) than the distal O(2) (−0.21e), reflecting the spatial polarization of the end-on configuration. In contrast to Configuration 1, both Configurations 2 and 3 exhibit substantially larger Fe–O bond populations (0.29 and 0.28, respectively), which is consistent with their more negative adsorption energies (−69.76 and −60.76 kJ/mol) and indicative of stronger Fe–O chemical anchoring. The O–O bond populations are 0.39 and 0.38, and the corresponding bond lengths are 1.278 and 1.287 Å. Both bonds are longer than that in gas-phase O_2_ (1.221 Å), consistent with O–O activation.

The DOS and PDOS of the three adsorption configurations are shown in Figure 3. The overlapping Fe 3d and adsorbed O 2p features below and near the Fermi level are consistent with Fe–O hybridization. The EDD maps and negative net Mulliken charges of the adsorbed O_2_ molecules support electron uptake from the surface, while O–O bond elongation and the calculated bond populations characterize the accompanying bond weakening.

**Figure 3:**
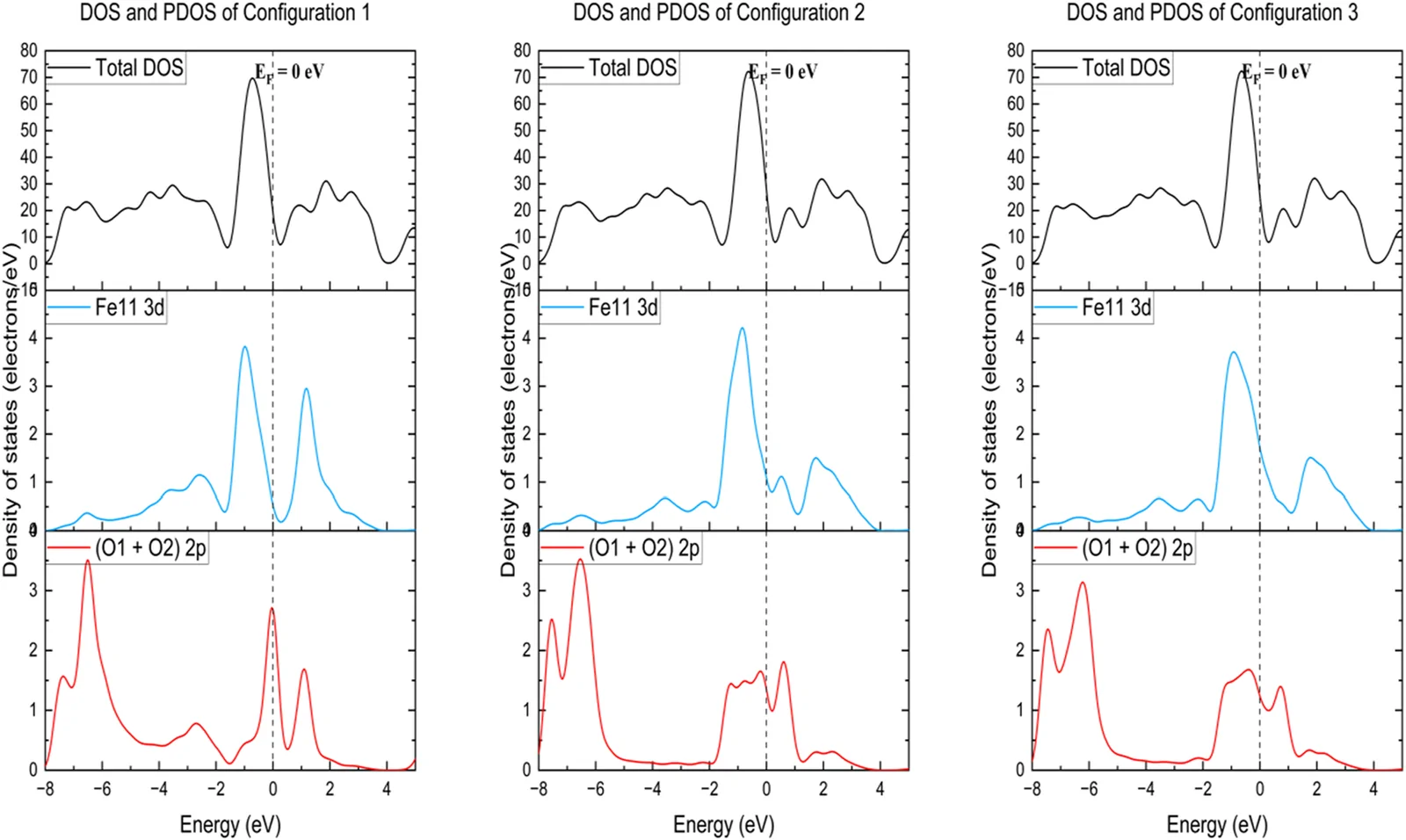
Total density of states (DOS) and projected density of states (PDOS) of the coordinated surface Fe 3d states and the combined 2p states of the two adsorbed O atoms in Configuration 1, Configuration 2, and Configuration 3. The Fermi level is set to 0 eV.

Taken together, the EDD, PDOS, and Mulliken analyses indicate that surface Fe sites mediate the initial molecular adsorption and activation of O_2_ in the configurations examined. No S–O bond formation was observed at this initial molecular-adsorption stage. The comparison also shows that adsorption stability and O–O activation do not vary in parallel: Configuration 2 is the most stable adsorption state, whereas Configuration 1 exhibits the greatest O–O elongation. This difference reflects the combined influence of Fe–O bonding, charge redistribution, and adsorption geometry.

### 3.2 Comparative Energy Profiles of O_2_ Dissociation Pathways

Nine O_2_ dissociation pathways were examined to compare O–O cleavage and the formation of Fe–O and S–O bonds. Previous studies have shown that the reactivity of S_3_c is significantly higher than that of S_4_c.^40^ Accordingly, this research focuses on two types of adsorption sites: Fe–S_3_c heteronuclear and S_3_c-S_3_c homonuclear adsorption. Geometric optimization yields four stable dissociated preoxidation adsorption configurations (A–D), whose electronic structures and bonding characteristics are shown in Figure 4.

**Figure 4:**
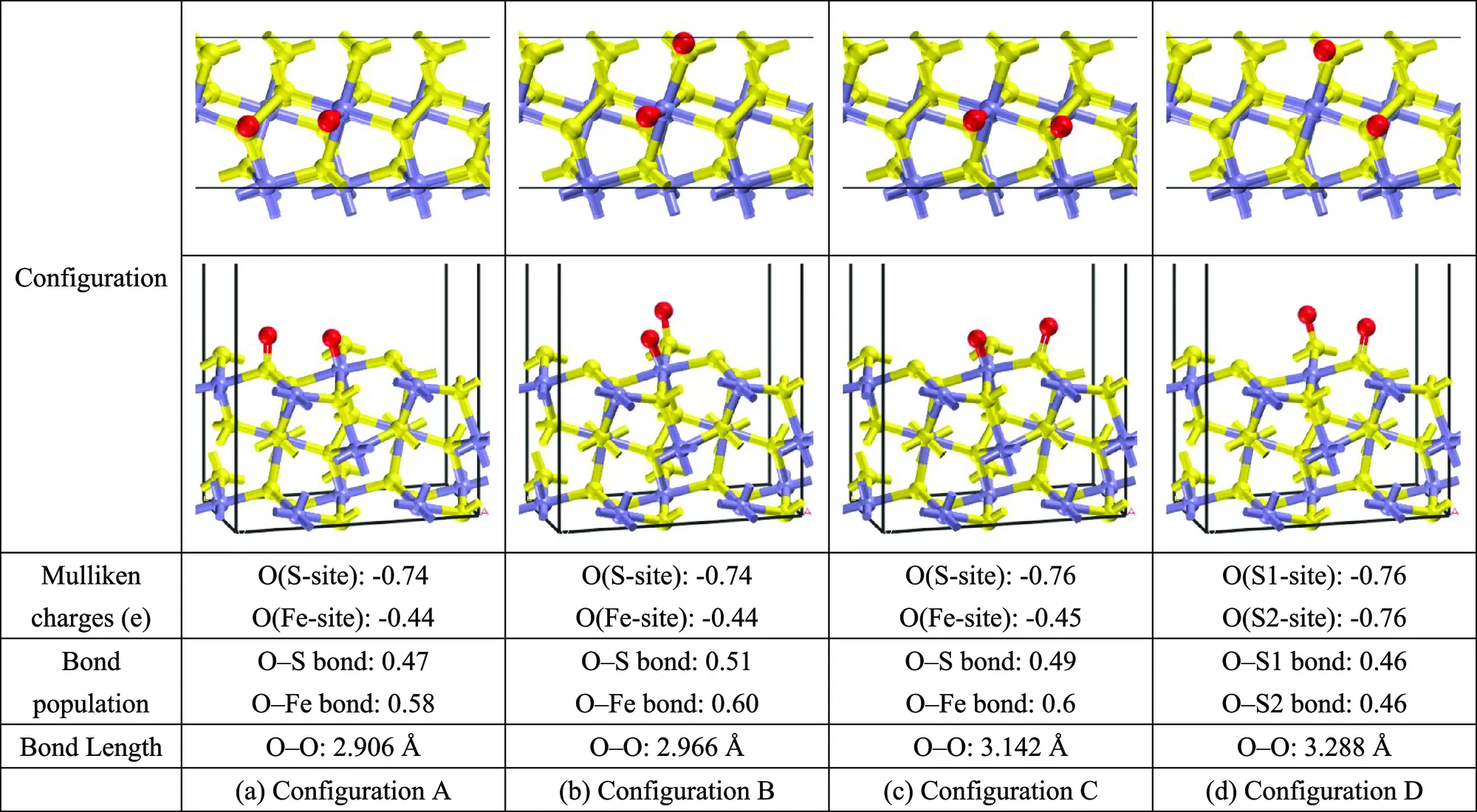
Electronic structures and bonding characteristics of the four stable dissociated preoxidized configurations on the pyrite (100) surface.

Mulliken charge analysis shows that in Configurations A–D, oxygen atoms adsorbed at S sites carry charges of −0.74e to −0.76e, significantly more negative than those at Fe sites (−0.44e to −0.45e). This indicates greater electron accumulation on the S-bound O atoms. The calculated Fe–O and S–O bond populations are 0.58–0.60 and 0.46–0.51, respectively, indicating bonding interactions between adsorbed O and both surface species. These structures show that surface S participates directly in the dissociation products through S–O bond formation.

To investigate the dissociation mechanism of O_2_ from adsorbed states (Configurations 1–3) to dissociated states (Configurations A–D), transition-state candidates were searched for the possible reaction pathways using the LST/QST method. Pathways that yielded unstable end points or failed to connect the intended initial and final states during the LST/QST search (e.g., 2→A, 2→D, 3→D) were excluded, yielding nine dissociation pathways for comparison. The LST/QST-derived transition-state-candidate structures and estimated barriers are shown in Figure 5.

**Figure 5:**
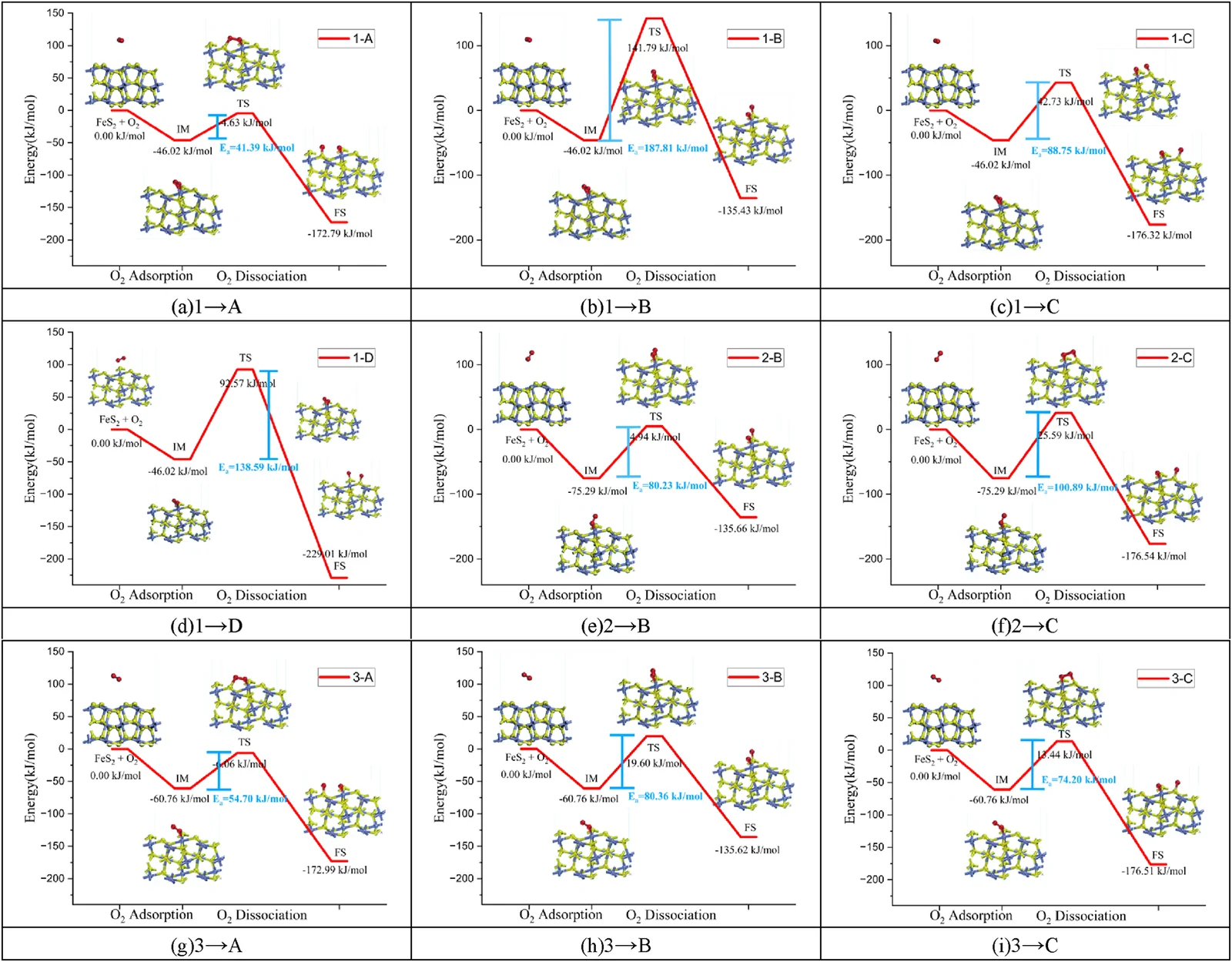
Estimated energy profiles for the O_2_ dissociation pathways on the pyrite (100) surface (IM = intermediate state, TS = transition-state candidate, FS = final state). Note: In each panel, the energy of the clean surface plus isolated O_2_ in the corresponding data set is set to zero. For Configuration 2, the adsorption calculation in Figure 1 and the dissociation profiles in this figure use the same isolated-system energy reference but different initial-state total energies, giving relative energies of −69.76 and −75.29 kJ/mol, respectively. The estimated barriers for 2→B and 2→C are calculated relative to the initial state used in their dissociation profiles.

Yang et al.^40^ reported an O_2_ dissociation barrier of 77.38 kJ/mol for their selected adsorption configuration. This value falls within the range obtained here (41.39–187.81 kJ/mol) and is close to those of pathways 2→B (80.23 kJ/mol), 3→B (80.36 kJ/mol), and 3→C (74.20 kJ/mol). The broader range obtained in the present study is associated with the different initial adsorption geometries and O–O cleavage channels considered within the same computational framework. Therefore, these values are used primarily to compare the relative competitiveness of different O_2_ dissociation pathways in this work. Absolute comparison with Yang et al. should also account for differences in surface supercell, slab-relaxation scheme, dispersion correction, cutoff energy, k-point sampling. Dispersion correction and spin treatment are not explicitly specified in Yang et al.’s Computational Methods section. In addition, the barrier of 197.96 kJ/mol reported by Yang et al.^40^ corresponds to the subsequent oxidation of a surface lattice S atom along IM1→TS1→IM2 in their Figure 6a.

**Figure 6:**
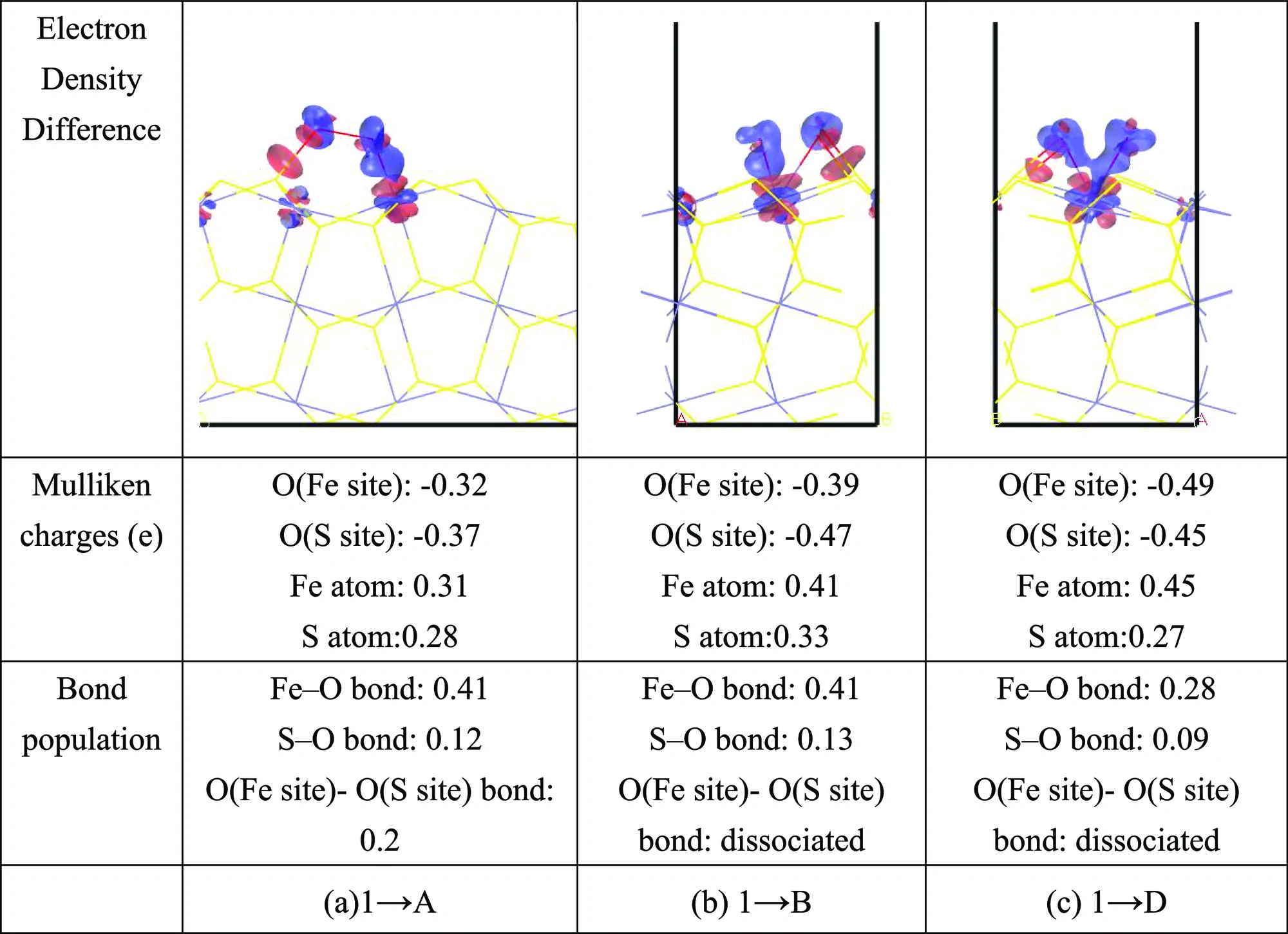
EDD and bonding characteristics of representative transition-state candidates for O_2_ dissociation on the pyrite (100) surface.

Path 1→A has the lowest estimated local barrier. Path 3→A has a higher local estimated barrier than path 1→A (54.70 versus 41.39 kJ/mol), but starts from a more stable adsorbed state. Its intermediate and transition-state candidate lie at lower energies relative to the clean surface and gas-phase O_2_ (IM: −60.76 versus −46.02 kJ/mol; TS candidate: −6.06 versus −4.63 kJ/mol). Thus, path 1→A has the lower barrier from its adsorbed intermediate, whereas path 3→A combines a more stable precursor with a comparably low transition-state-candidate energy. The dissociation steps for 1→A, 1→D, and 3→A are all exothermic, with reaction energies of −126.77, −182.99, and −112.23 kJ/mol, respectively.

To examine the electronic and bonding differences among these pathways, 1→A, 1→D, and 1→B were selected as representative low-, intermediate-, and high-barrier cases for EDD and bonding analysis, as shown in Figure 6. This analysis aims to clarify how electronic structure favors O–O cleavage through combined Fe–O and S–O interactions.

The transition-state candidate of the low-barrier pathway 1→A exhibits an Fe–O–O–S bridging configuration. In this candidate structure, the two O atoms have Mulliken charges of −0.32e and −0.37e, while the coordinated Fe and S atoms have charges of +0.31 and +0.28 e, respectively. The net charge of −0.69e on the O atom pair is consistent with electron uptake from the surface. This process significantly elongates the O–O bond to 1.53 Å (compared to 1.221 Å in the gas phase) and reduces its bond population to 0.20, substantially weakening (not fully breaking) the covalent bond. The positive Fe–O and S–O bond populations of 0.41 and 0.12 indicate simultaneous bonding interactions with the two surface sites.

In contrast, the transition-state candidates of pathways 1→B and 1→D exhibit O–O distances of 1.722 Å and 1.802 Å, respectively, indicating substantially greater O–O separation than in 1→A. In both cases, one O atom binds to the Fe site while the other interacts with a neighboring S atom. The two O atoms carry Mulliken charges of −0.39e and −0.47e in 1→B and −0.49e and −0.45e in 1→D. However, the Fe–O and S–O bond populations in 1→D (0.28 and 0.09) are lower than those in 1→A (0.41 and 0.12), indicating weaker local bonding. Pathway 1→A therefore combines a strongly elongated but incompletely cleaved O–O bond with simultaneous Fe–O and S–O interactions. These comparisons suggest that the barrier differences reflect the combined effects of O–O distortion and local Fe–O–S coordination rather than charge distribution alone.

### 3.3 O_2_ Readsorption Behavior on the Preoxidized Surface

Adsorption of a second O_2_ molecule was examined on the preoxidized Configuration A. Initial models with O_2_ in end-on and side-on configurations were constructed at available active sites – including adjacent Fe sites, S_3_c sites, and hollow sites – and were subsequently geometrically optimized, yielding six geometry-optimized structures, as shown in Figure 7.

**Figure 7:**
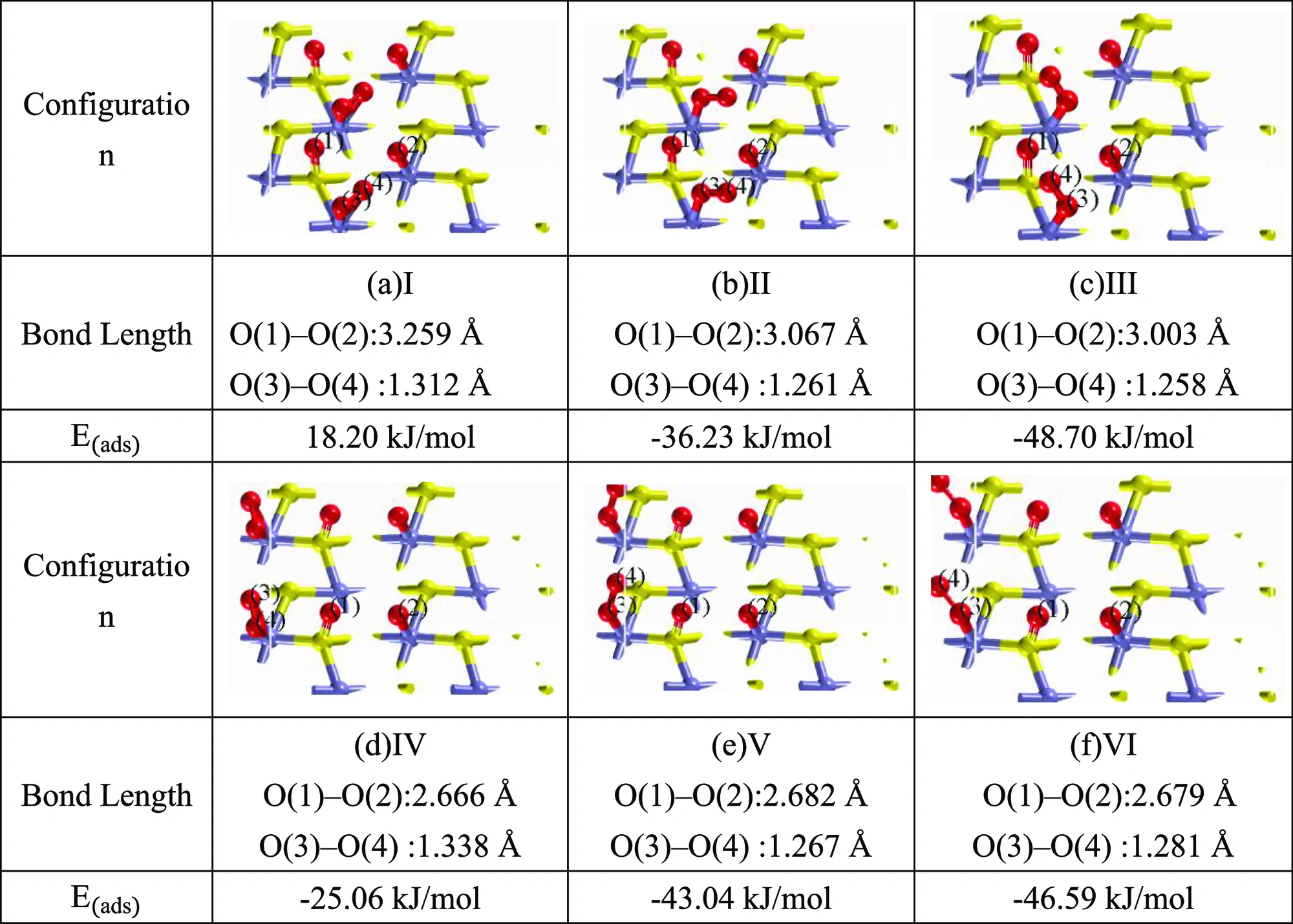
Geometry-optimized readsorption configurations and key parameters of the second O_2_ molecule on the preoxidized pyrite (100) surface.

Configuration I has a positive adsorption energy (+18.20 kJ/mol), indicating that adsorption is energetically unfavorable relative to the adopted reference state. Among the remaining configurations, the strongest adsorption is observed for Configuration III (−48.70 kJ/mol), which is still considerably weaker than that of the most stable adsorption state on the pristine surface (Configuration 2, −69.76 kJ/mol). The adsorption stability of the second O_2_ molecule follows the order: III > VI > V > II > IV > I.

These readsorption configurations are all built upon the premise that the first O_2_ molecule has dissociated, forming the S–O(1) and Fe–O(2) functional groups on the surface, with S–O(1) serving as the core framework for subsequent SO_2_ construction.

Configurations I and IV adopt side-on adsorption geometries, whereas II, III, V, and VI are end-on. These geometries differ in the positions and coordination of the O atoms available for subsequent S–O bond formation.

These differences in adsorption geometry may lead to different structural reorganization requirements in the subsequent precursor formation steps.

### 3.4 Competing Pathways for SO_2_ Precursor Formation and Desorption

This section identifies potential SO_2_ precursors (O–S–O configurations) through geometry optimization and evaluates their relative feasibility using LST/QST-derived energy profiles. Configuration I exhibits positive readsorption energy, indicating energetically unfavorable O_2_ readsorption under the present reference state; therefore, we did not further explore subsequent reaction pathways originating from this configuration.

Among the derivatives of configurations II and IV, only II-(2) and IV-(2) provided continuous LST/QST pathways with a transition-state candidate. Configurations V and VI yield two precursor candidates. Consequently, a total of six pathways with complete LST/QST-derived reaction profiles, including transition-state candidates and estimated barriers, were obtained.

Although Configurations II and III share similar initial end-on adsorption geometries, the different orientation of the Fe–O(3)–O(4) bond allows Configuration II to form the O–S–O–Fe precursor structure (II-(2)) via O(3). No analogous precursor was obtained from Configuration III in the searches performed.

Configuration IV has an O(3)–O(4) distance of 1.338 Å. In the IV-(2) pathway, O(4) forms a bond with the surface S atom to produce an O–S–O precursor.

Configurations V and VI both adopt end-on adsorption modes and can form precursors through two alternative oxygen atoms. For Configuration V, the newly adsorbed O(4) can directly participate in bonding to form the O(4)–S–O(1) structure in V-(1); meanwhile, O(2) from the original Fe–O(2) is able to form the O(2)–S–O(1)–Fe structure in V-(2). For Configuration VI, the newly adsorbed O(3) participates in bonding to form the O(3)–S–O(1) structure in VI-(1), while its original O(2) can similarly transfer to form the O(2)–S–O(1) structure in VI-(2).

As shown in Figure 8, II-(2) and V-(2) adopt O–S–O-Fe structures, with upward S atom displacements of 0.921 and 0.302 Å, respectively. In contrast, IV-(2), V-(1), VI-(1), and VI-(2) contain surface-bound O–S–O units and exhibit S atom vertical displacements below 0.1 Å.

**Figure 8:**
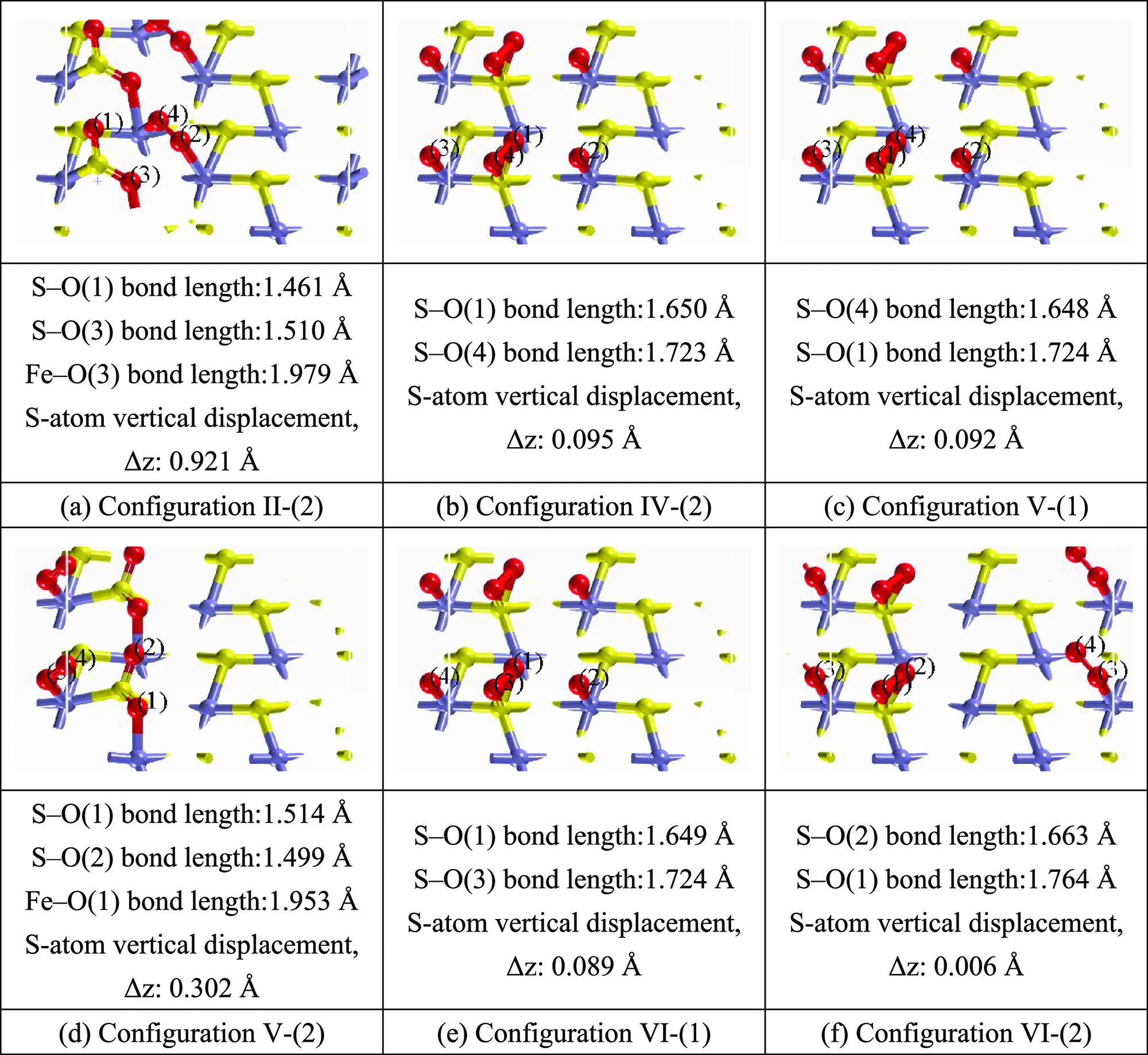
Stable configurations of SO_2_ precursors and structural parameters on the preoxidized pyrite (100) surface. Note: Δ*z* denotes the displacement of the reacting S atom along the surface normal relative to its position in the corresponding readsorbed configuration.

The two precursors incorporate O atoms from different adsorption steps. In II-(2), O(3) from the second adsorbed O_2_ molecule forms an S–O bond, with Fe–O(3) coordination retained in the precursor. In V-(2), O(2) produced by the initial O_2_ dissociation participates in forming the O–S–O–Fe structure.

To complement the precursor structures shown in Figure 8, representative transition-state candidate structures of II-(2) and V-(2) were further examined, as shown in Figure 9. In the II-(2) transition-state candidate, the surface S atom is lifted markedly from the surface, with an S atom vertical displacement of 0.556 Å. The S atom remains bonded to O(1) with an S–O(1) distance of 1.514 Å, while the approaching O(3) is located at 1.993 Å from S. Meanwhile, the O(3)–O(4) distance is elongated to 1.602 Å, and O(3) and O(4) remain coordinated with Fe, with Fe–O distances of 1.882 and 1.877 Å, respectively. These structural features indicate that II-(2) involves simultaneous S lifting, O–O weakening, and extensive local rearrangement.

**Figure 9:**
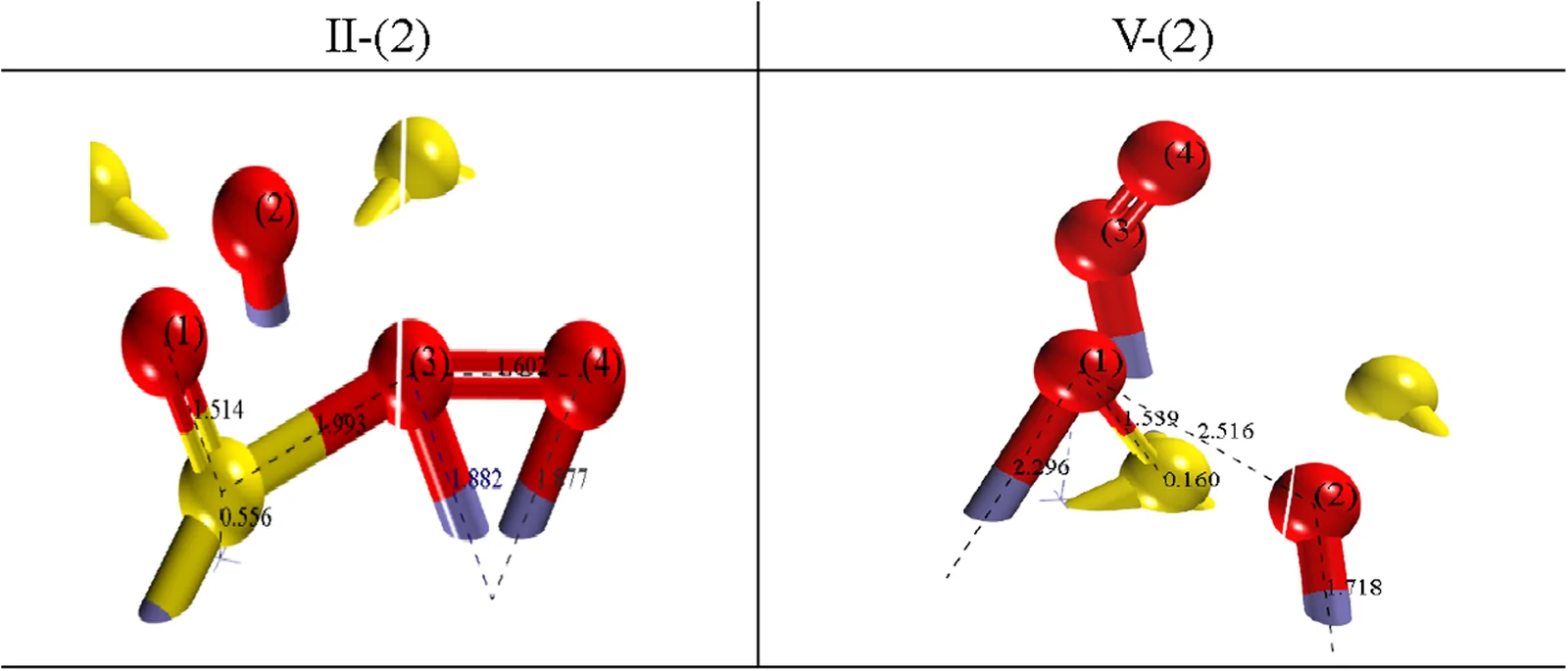
Representative transition-state candidate structures for SO_2_ precursor formation along II-(2) and V-(2). Distances are given in Å. Δz denotes the displacement of the reacting S atom along the surface normal relative to its position in the corresponding readsorbed configuration, II or V.

The V-(2) transition-state candidate shows a smaller S atom vertical displacement of 0.160 Å compared with 0.556 Å for II-(2), and the target O(2) atom retains Fe–O coordination at 1.718 Å. The smaller S displacement is consistent with the lower estimated precursor-formation barrier of V-(2) relative to II-(2).

Table 1 summarizes the estimated barriers and reaction energies for the examined steps, while Figure 10 compares the six SO_2_ precursor-formation profiles.

**Figure 10:**
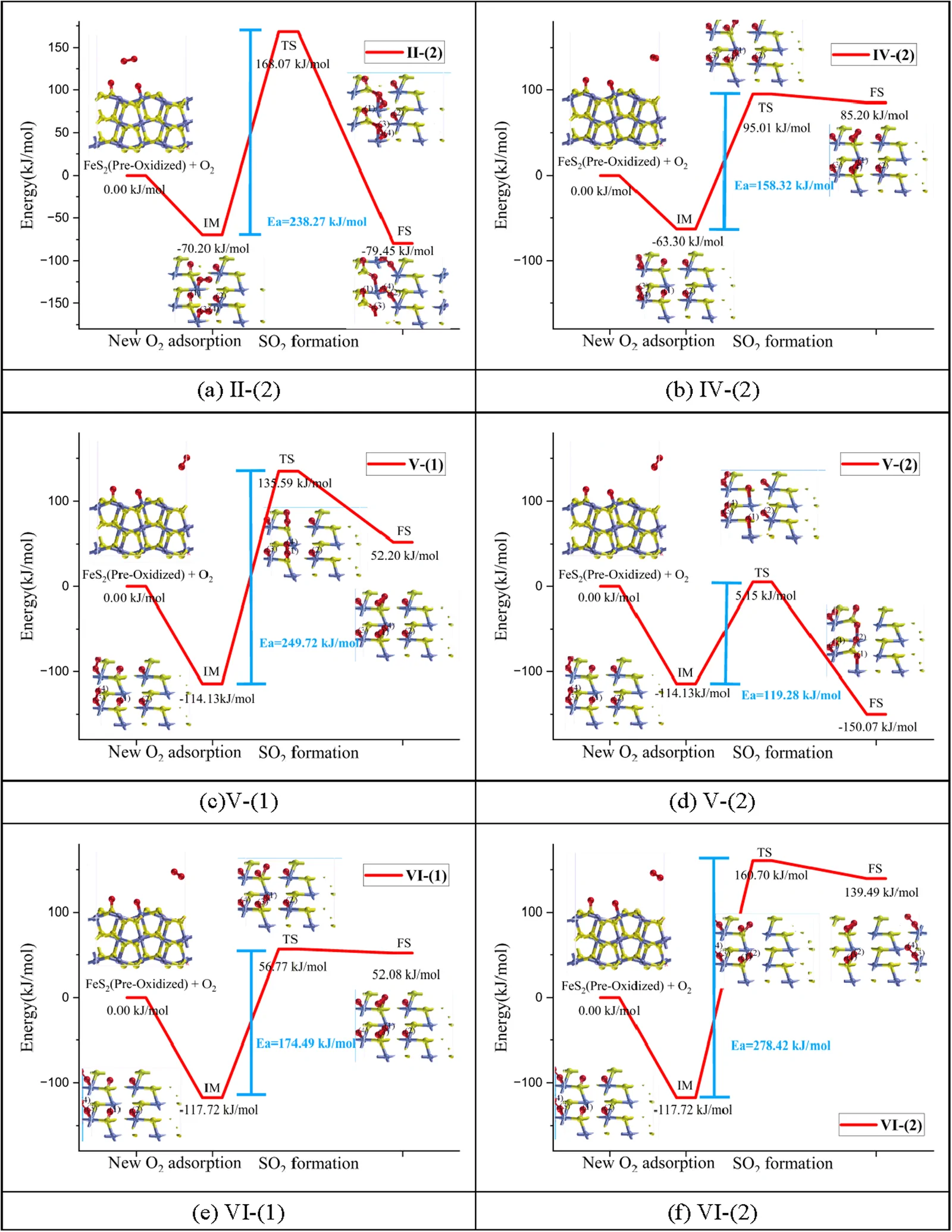
LST/QST-derived estimated energy profiles for SO_2_ precursor formation on the preoxidized pyrite (100) surface. Note: Energies in each panel are referenced to the corresponding “before adsorption” configuration in the pathway-energy data set. This reference differs from the sum of the separately optimized surface and O_2_ energies used to calculate the adsorption energies in Figure 7. The IM coordinates in this figure therefore use a different energy reference from the adsorption energies in Figure 7. IM, TS, and FS denote the readsorbed intermediate, transition-state candidate, and SO_2_ precursor, respectively. The local estimated barrier and reaction energy are calculated as *E*_a_ = *E*_TS_ – *E*_IM_ and Δ*E* = *E*_FS_ – *E*_IM_.

**Table 1:** LST/QST-Derived Estimated Barriers and Reaction Energies for the Examined Reaction Steps (kJ/mol)

initial O_2_ dissociation			SO_2_ precursor formation			SO_2_ desorption		
	*E*_a_	Δ*E*		*E*_a_	Δ*E*		*E*_a_	Δ*E*
1→A	41.39	–126.77	II-(2)	238.27	–9.25	II-(2)	83.88	+80.34
1→B	187.81	–89.41	IV-(2)	158.32	+148.50	IV-(2)	242.07	–195.01
1→C	88.75	–130.30	V-(1)	249.72	+166.33	V-(1)	240.13	–195.09
1→D	138.59	–182.99	V-(2)	119.28	–35.94	V-(2)	112.75	+34.30
2→B	80.23	–60.37	VI-(1)	174.49	+169.80	VI-(1)	239.70	–194.81
2→C	100.89	–101.25	VI-(2)	278.42	+257.21	VI-(2)	280.31	–185.24
3→A	54.70	–112.23						
3→B	80.36	–74.86						
3→C	74.20	–115.75						

The estimated precursor formation barriers range from 119.28 to 278.42 kJ/mol (Figure 10). V-(2) has the lowest value, referenced to the readsorbed configuration V intermediate. The local barrier for II-(2) is 238.27 kJ/mol.

As summarized in Table 1, pathway II-(2) has the lowest estimated SO_2_-desorption barrier (83.88 kJ/mol), whereas pathway VI-(2) has the highest value (280.31 kJ/mol). Among these routes, V-(2) shows relatively low estimated barriers for both SO_2_ precursor formation and subsequent SO_2_ desorption with values of 119.28 and 112.75 kJ/mol, respectively.

To further characterize the electronic evolution of this representative pathway, end point population results for the readsorbed Configuration V and the SO_2_-separated final state are summarized in Table 2.

**Table 2:** Population Charges from the Re-Adsorbed Configuration V (Initial) to the SO_2_-Desorbed Final State along the V-(2) Pathway (Unit: e)^a^

atomic group	Mulliken initial	Mulliken final	Mulliken Δ*q*	Hirshfeld initial	Hirshfeld final	Hirshfeld Δ*q*
SO_2_-forming atom group	–0.370	–0.008	+0.362	–0.19	–0.02	+0.17
residual surface O–O group	–0.279	–0.701	–0.422	–0.09	–0.21	–0.12
all O atoms	–1.415	–2.324	–0.909	–0.49	–0.64	–0.15
all S atoms	+0.681	+1.473	+0.792	+1.45	+1.52	+0.07
all Fe atoms	+0.749	+0.864	+0.115	–0.95	–0.88	+0.07

a Note: Δ*q* = *q*_final_ – *q*_initial_. A negative Δq denotes electron accumulation, whereas a positive Δ*q* denotes electron depletion. Because population charges depend on the selected partitioning scheme, emphasis is placed on the direction and relative magnitude of the end point changes. The atom-group rows overlap with the element totals and should not be summed together.

Between the readsorbed Configuration V and the SO_2_-separated final state, the SO_2_-forming atom group changes its Mulliken charge from −0.370e to −0.008e. Within this group, the S atom loses 0.849e and the two O atoms gain 0.487e, yielding a net charge change of +0.362e. The final SO_2_ molecule is therefore nearly neutral. The remaining two O atoms gain 0.422e over the same end point comparison.

Summed over each element, the Mulliken charge changes are −0.909e for O, +0.792e for S, and +0.115e for Fe. Hirshfeld analysis gives changes of −0.15e, +0.07e, and +0.07e, respectively, supporting the same direction of net charge redistribution.

## 4 Limitations and Future Work

The reported energies are static electronic energies without zero-point or thermal corrections, and the LST/QST-derived structures are treated as transition-state candidates because vibrational frequency and intrinsic reaction coordinate analyses were not included. Therefore, the calculated barriers are used for a comparative mechanistic discussion among the modeled pathways.

The initial adsorption models contain one O_2_ molecule per (2 × 1) surface supercell. A second O_2_ molecule is introduced onto the preoxidized surface in the readsorption models, giving four O atoms in the subsequent reaction models. Coverage was not varied systematically, and alternative surface terminations, pre-existing defects, and water coadsorption were not examined. These factors may alter adsorption geometries, intermediate stability, and the relative barriers of competing pathways. Future work should extend the comparison to these conditions using verified transition states and consistently referenced free-energy profiles.

## 5 Conclusions

This study compares the O_2_ activation and SO_2_ formation pathways on an idealized pyrite (100) surface using DFT calculations. The main findings are summarized as follows.

First, stable molecular O_2_ adsorption was obtained only at Fe sites in the configurations examined. Fe–O hybridization, electron accumulation on O_2_, and O–O bond elongation characterize its adsorption and activation.

Second, O_2_ dissociation proceeds through multiple competing pathways, with LST/QST-derived estimated barriers ranging from 41.39 to 187.81 kJ/mol. Among these pathways, the 3→A route shows a relatively favorable balance between adsorption stability and dissociation barrier, suggesting a representative candidate pathway for molecular oxygen activation on the pyrite (100) surface.

Third, among the six SO_2_ precursor formation pathways and their subsequent desorption steps, V-(2) shows relatively low estimated barriers for both precursor formation and subsequent SO_2_ desorption, with values of 119.28 and 112.75 kJ/mol, respectively.

Figure 11 summarizes the representative sequence 3→A→V→V-(2) assembled from the comparisons within individual reaction stages.

**Figure 11:**
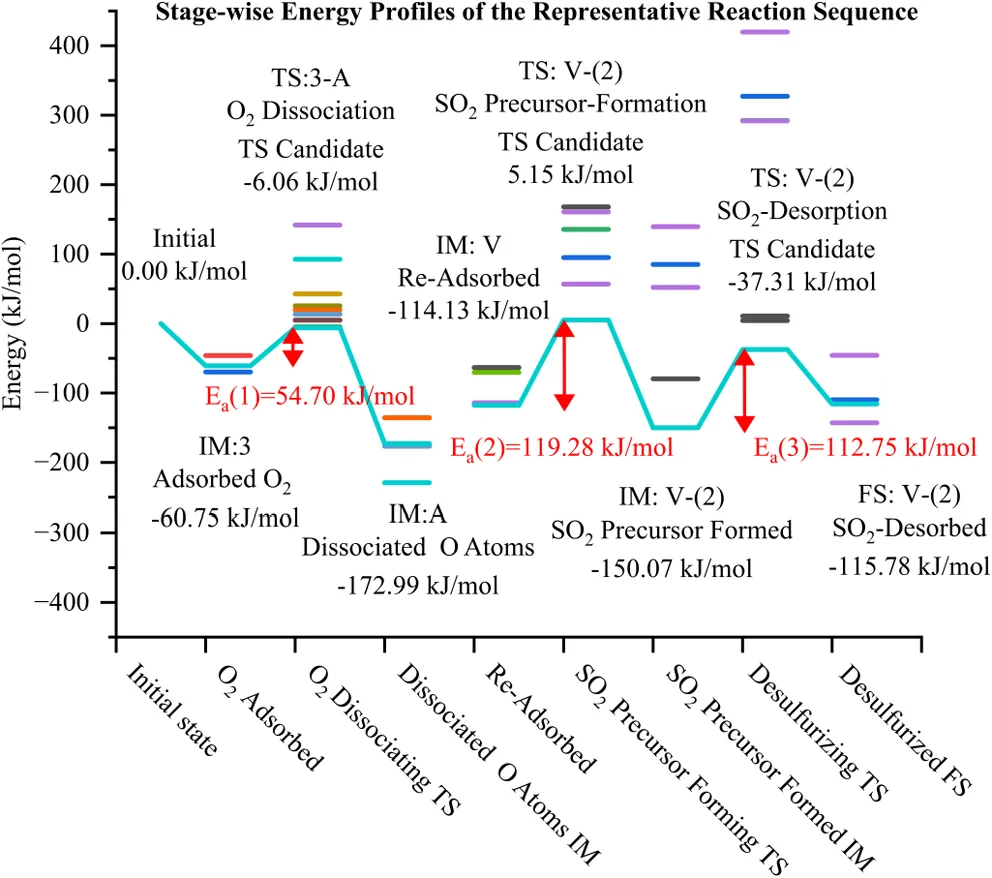
Representative reaction sequence assembled from comparisons within individual stages. The indicated barriers are referenced to the initial state of each corresponding step. Note: The three elementary steps retain their respective pathway-specific energy references. Therefore, the connecting lines and vertical offsets between stages are schematic and should not be interpreted as a single continuous absolute-energy profile.

## Supplementary Material



## Notes

Cutoff-energy sensitivity test (Figure S1) (PDF)
