# Supplementary material for: Comparative Pathways of SO2 Formation on the Pyrite (100) Surface: A DFT Study of O2 Activation and Surface Sulfur Oxidation
Source: ACS Omega. 2026 Oct 23;11(43):66712–23. doi: 10.1021/acsomega.6c06241 (PMC13736855; doi:10.1021/acsomega.6c06241)
Supplement: Supplementary file 1 [file ao6c06241_si_001.pdf]

Supporting Information

Comparative Pathways of SO_2 Formation on the Pyrite (100) Surface: A DFT Study of O_2 Activation and Surface Sulfur Oxidation

Mingrui Liu[1], Hongtao Li[2], Xiqing Xu[3], Jie Sha[1]*, Guangyuan Xie[1]

1. Key Laboratory of Coal Processing and Efficient Utilization of Ministry of Education, School of Chemical Engineering and Technology, China University of Mining and Technology, Xuzhou 221116, China;
2. Chifeng Disaster Prevention and Relief Center, Chifeng 024000, China;
3. State Key Laboratory of Continental Shale Oil, Daqing 163712, China;

* Correspondence: shajie@cumt.edu.cn; Tel.: +86-13813469381

S1. Cutoff Energy Validation

The sensitivity of the total energy to the plane-wave cutoff energy was examined using a representative 38-atom model. The cutoff energy was varied from 450 to 700 eV, while the other computational settings were kept unchanged. As shown in Figure S1, the energy variation per atom decreased progressively with increasing cutoff energy and became relatively small above 550 eV. The CASTEP "Fine" setting of 571.40 eV was therefore retained as a balance between numerical accuracy and computational cost.

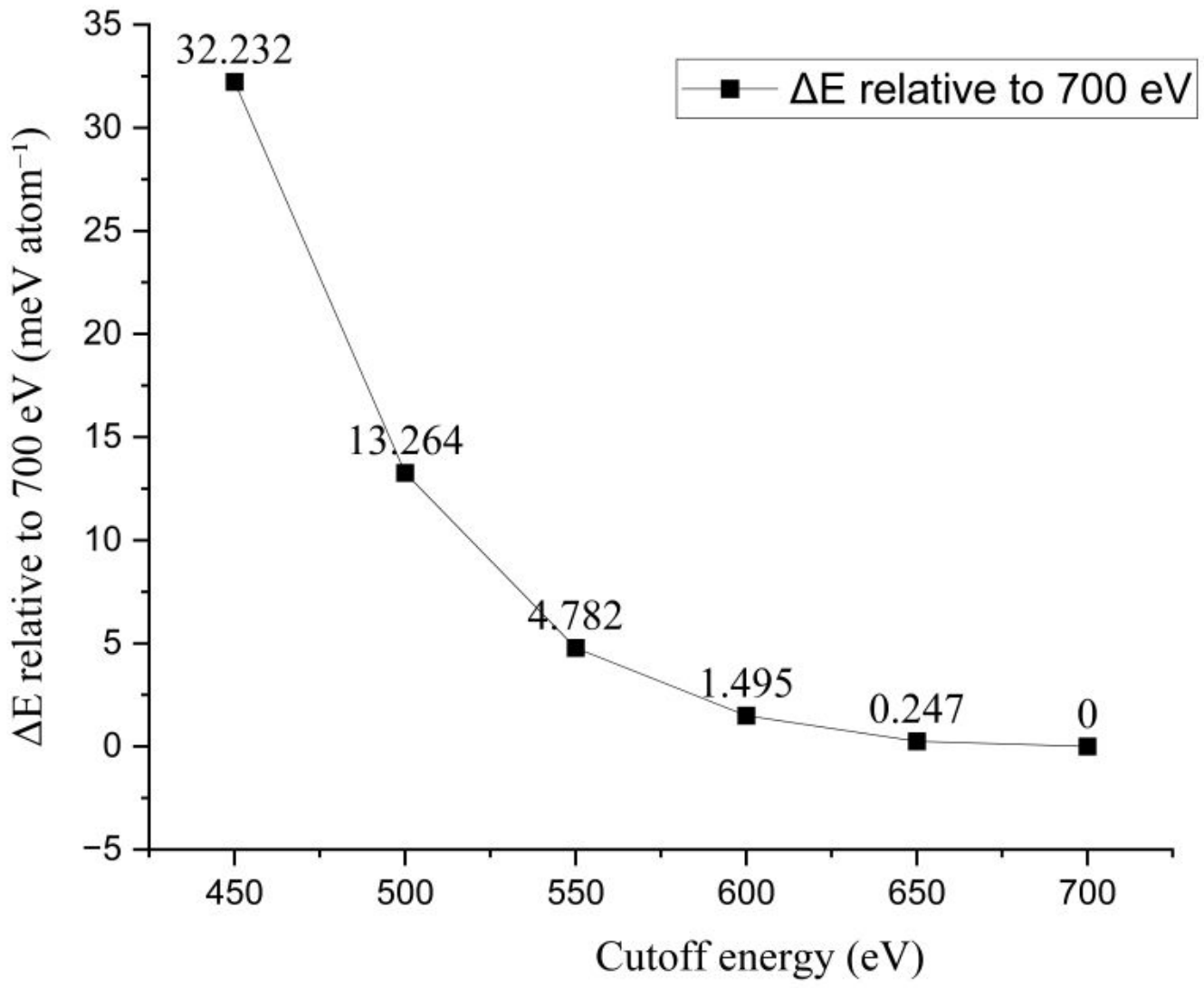

Figure S1. Convergence of the dispersion-corrected total energy per atom with respect to the plane-wave cutoff energy for the representative 38-atom model.
